# The Trend, Characteristics and Treatment Outcomes in Patients with Tuberculosis Undergoing Thoracic Surgery in the Kyrgyz Republic between 2017 and 2021

**DOI:** 10.3390/tropicalmed8080393

**Published:** 2023-07-31

**Authors:** Konushbek Sakmamatov, Yulia Kuznetsova, Kylychbek Istamov, Daniil Shauer, Jaya Prasad Tripathy, Anthony D. Harries, Kudaibergen Osmonaliev, Olga Goncharova

**Affiliations:** 1 Faculty of Medicine, Ala-Too International University, Bishkek 720000, Kyrgyzstan; ksakmamatov@gmail.com (K.S.); kudaibergen.osmonaliev@alatoo.edu.kg (K.O.); 2 International Charitable Foundation “Alliance for Public Health”, 01601 Kiev, Ukraine; 3 School of Medicine, Osh State University, Osh 723500, Kyrgyzstan; edu@oshsu.kg; 4 National Centre of Phthisiology, Ministry of Health, Bishkek 720000, Kyrgyzstan; daniilshauer@gmail.com (D.S.); goncharova.ncph@gmail.com (O.G.); 5 Department of Community Medicine, All India Institute of Medical Sciences, Nagpur 441108, India; jtripathy@aiimsnagpur.edu.in; 6 International Union Against Tuberculosis and Lung Disease, 2 Rue Jean Lantier, 75001 Paris, France; adharries@theunion.org; 7 Department of Clinical Research, London School of Hygiene and Tropical Medicine, Faculty of Infectious and Tropical Diseases, Keppel Street, London WC1E 7HT, UK

**Keywords:** Kyrgyzstan, tuberculosis, drug-resistant tuberculosis, thoracic surgery, tuberculoma, pleural empyema, surgical treatment outcomes, TB treatment outcomes, SORT IT, operational research

## Abstract

Surgery has played an important role in managing complicated tuberculosis in former Soviet Union countries, including the Kyrgyz Republic. However, published information is limited. This study aimed to document the trend, characteristics and outcomes of tuberculosis patients who underwent thoracic surgery, using routinely collected data. Between 2017 and 2021, 4–7% of tuberculosis patients in the Kyrgyz Republic underwent thoracic surgery in two centres in Bishkek and Osh. In 2021, case records were retrieved in 264 (78%) of 340 patients undergoing thoracic surgery in the country. The most common indications for surgery were pleural exudate/empyema in 127 (44%) and tuberculoma in 83 (32%). Most patients (73%) underwent surgery within 30 days of starting TB treatment. Two-thirds of patients underwent radical surgery, and surgical outcomes were excellent in 99% of patients with one death. Post-operatively, 63 (23%) patients had no TB detected by the histology, with the two most common specified conditions being lung cancer and pulmonary hydatid disease. TB treatment was stopped in these patients. Of the 201 patients with confirmed TB after surgery, TB-treatment success was documented in 163 (81%), died/failure/lost to follow-up in 10 (5%) and not evaluated in 28 (14%). This study shows that thoracic surgery is feasible, safe and effective in the routine programme setting. Recommendations are made to strengthen referral and monitoring systems.

## 1. Introduction

Despite the advances in the prevention, diagnosis and treatment of tuberculosis (TB) in the last few decades, the disease remains a global public health threat and is one of the largest killers out of all infectious diseases worldwide. Since 2020, the COVID-19 pandemic has resulted in a severe disruption in TB services, with, for the first time in many years, an increase in the number of people falling ill with TB and drug-resistant TB (DR TB). In 2021, an estimated 10.6 million people fell ill with TB, 450,000 developed rifampicin-resistant TB and 1.6 million people died from TB [1].

Although new anti-TB drugs have been developed and are being incorporated into anti-TB drug regimens, particularly for DR TB, surgery has, and continues to play, an important role in the management and treatment of complex cases. The main indications for surgery are the (i) elimination of persisting infectious cavities despite chemotherapy, (ii) resection of destroyed lung tissue, (iii) resection of tuberculomas and (iv) treatment of pleural empyema. The overall TB treatment success rates for those undergoing surgical treatment are reported to range from 75 to 98% [2]. Massive haemoptysis, resulting from large TB cavities or aspergillomas infecting TB cavities, is another important indication for surgery. In a series of patients with massive haemoptysis, 70% of whom had either active or inactive TB, mortality was significantly reduced with medical treatment and pulmonary resection compared with medical management alone [3].

With generally poor treatment success rates for the chemotherapy of multidrug-resistant (MDR) TB, surgery has also re-emerged as a successful adjuvant therapeutic strategy. A systematic review and meta-analysis showed a treatment success rate of 82% with surgery compared with 60% without surgery [4]. While there are no randomised controlled trials on the efficacy of surgery in the treatment of TB, there has been an international review of existing practices and expert opinion, which has provided guidance on indications and contraindications for surgery, the timing of surgery, the types of surgical intervention and post-operative management [5].

Surgery has always played an important role in the management of TB in former Soviet Union countries. For example, in Georgia, Eastern Europe, favourable outcomes and low complication rates were associated with surgery for both drug-susceptible (DS) and DR pulmonary TB [6,7]. In Uzbekistan, Central Asia, about 10% of diagnosed patients underwent surgical interventions as an adjunct to anti-TB chemotherapy, with low rates of post-operative complications and high rates of treatment success above 90% [8]. 

The Kyrgyz Republic is one of the high-burden TB and DR TB countries in the WHO European Region, and, along with seven other countries in Eastern Europe and Central Asia, has seen a gradual increase in notification rates of DR TB in the last two decades [9]. The most recent national guidelines on the management of DR TB emphasize the role of surgical treatment as an adjunct to anti-TB chemotherapy (Ministry of Health Kyrgyzstan. Clinical guidance on management of drug resistant tuberculosis. 2020). There are two clinics in the Kyrgyz Republic that provide thoracic surgery for pulmonary TB patients: Bishkek clinic, operating on about 200–300 patients per year; and Osh clinic, operating on about 50–100 patients per year.

Despite this surgical work, there has been no published study in the country documenting the proportion of TB patients undergoing surgery in the last few years or determining the characteristics of patients being operated on, the indications for surgery, the surgical procedures themselves and the surgical and TB treatment outcomes. Such information will be valuable for the Kyrgyz Republic National TB Programme to assess the role of surgery during the COVID-19 era, when health services were severely disrupted, and at a time when new drugs and new short-course regimens are becoming available for managing both DS TB and DR TB. It will also inform on the timing of surgery in relation to when anti-TB chemotherapy was started and whether additional safety measures are needed for surgical staff in the event of surgery taking place soon after anti-TB treatment, when the patient may still be infectious.

The aim of the study, therefore, was to determine the trend, characteristics and outcomes of all registered TB patients receiving thoracic surgery in the Kyrgyz Republic between 2017 and 2021. The specific objectives were to document the following: (1) between 2017 and 2021, the trend in the number and proportion of notified TB patients in the Kyrgyz Republic receiving thoracic surgical treatment (pre-COVID-19 and during COVID-19); and (2) in 2021, for all TB patients undergoing thoracic surgical treatment in the two clinics of Bishkek and Osh—(a) their sociodemographic and clinical characteristics, (b) the timing of surgery in relation to TB treatment, (c) details of the surgery, (d) TB treatment outcomes of those with confirmed TB after surgery had taken place and (e) factors associated with TB treatment success in those with confirmed TB.

## 2. Materials and Methods

### 2.1. Study Design

The study was performed in two parts: a longitudinal trend analysis over five years, from 2017 to 2021, using secondary routinely collected aggregate data; and a cohort analysis in 2021 from the two surgical clinics, using secondary routinely collected individual data.

### 2.2. Setting

#### 2.2.1. General Setting

The Kyrgyz Republic is a landlocked lower/middle-income country located in Central Asia; it borders Kazakhstan, Tajikistan, Uzbekistan and China. It has a population of about 6.7 million and a GDP per capita of USD 8683 [10]. The country is divided into seven regions, which are subdivided into 44 districts, with the two cities of Bishkek and Osh having state importance and not belonging to any particular region.

#### 2.2.2. National TB Epidemiology

The estimated TB incidence in the Kyrgyz Republic for 2021 was approximately 8500, with an incidence rate of 130 per 100,000. Rifampicin-resistant (RR)/MDR-TB incidence was 3200, with an incidence rate of 49 per 100,000 [1]. The number of new and previously treated TB case notifications for 2021 was 5199, giving a TB treatment coverage rate of 61%. The treatment success in new and relapsed TB patients enrolled in 2020 was 82%; for RR/MDR-TB patients enrolled in 2019, it was 72%; and for pre-XDR/XDR (extensively drug resistant) TB patients enrolled in 2019, it was 62% [1].

#### 2.2.3. National TB Control

There is a National TB Control Programme with the central unit (National Centre for Phthisiology) based in the Ministry of Health, Bishkek. Its main responsibilities are the country-wide coordination of TB control activities; oversight of the national TB programme, including strategic planning, national policy and guidelines; monitoring, evaluation and surveillance; and provision of specialised care for TB patients. Under the National Centre for Phthisiology, there is a second tier of 5 Republican PTB centres, 9 regional/city TB control centres, 3 TB hospitals and 1 Prison TB Hospital. These, together, serve 63 TB treatment cabinets in a third tier at regional centres of family medicine or centres of general medical practice.

Diagnosis and treatment of TB in the country is provided free of charge. There is a good laboratory network composed of a single National Reference Laboratory, which performs drug susceptibility testing using various methodologies, including line probe assays; and regional/district laboratories, which perform sputum smear microscopy and/or Xpert MTB/RIF assays (Cepheid, Sunnyvale, California, USA). Patients are diagnosed as having bacteriologically confirmed TB on the basis of smear microscopy and the Xpert MTB/RIF assay and are further stratified into drug-susceptible (DS) or drug-resistant (DR) TB patients. Patients with clinically diagnosed TB have this diagnosis made on the basis of clinical symptoms and signs, with chest radiography and computed tomography where available. With these radiographic and scanning techniques, tuberculoma is usually diagnosed on the basis of a solid lesion that is usually round; cirrhotic pulmonary/pleural disease, on the basis of fibrosis and pleural involvement; fibro-cavitary/cavernous disease, on the basis of fibrosis and cavitation; and pleural exudate and empyema, on the basis of a pleural effusion combined with the results of a pleural aspirate examining the fluid for protein, white blood cells and presence of mycobacterial TB on microscopy for acid-fast bacilli and/or Xpert MTB/RIF assays.

Patients diagnosed with TB may be hospitalised during the first months of treatment (the intensive phase) if there are specific indications, or they may be treated as outpatients. The continuation phase is usually managed in the outpatient departments. All patients with DS TB, new and previously treated, receive the WHO recommended standardised 6–8-month duration regimen, using first-line drugs [11]. The treatment of DR TB is initiated and managed by a Medical Concilium that is composed of experienced medical professionals located at regional TB centres. Treatment options are decided on a case-by-case basis and include individualised or short regimens that are in line with WHO Guidelines [12,13].

Patients are followed up during treatment at the nearest TB centre or cabinet via a clinical assessment, sputum smear microscopy or, in the case of DR TB, by monitoring sputum culture conversion for the first six months and at the end of treatment. There is a patient support programme to ensure proper adherence to treatment, and this includes transportation costs. Final treatment outcomes are reported in line with the WHO guidelines for DS TB and DR TB [14].

Programmatic data on case-finding and -treatment outcomes are collected on a monthly basis on paper-based cards and an electronic database. The data are checked and analysed by TB cabinet statisticians, and the data are submitted monthly to the “Foundation Obligatory Health Insurance”. Data are collated and reported annually to the National Centre of Phthisiology, Ministry of Health.

#### 2.2.4. Surgery for TB in the Kyrgyz Republic

There are two clinics for thoracic surgery (Bishkek and Osh) that serve pulmonary TB patients in the Kyrgyz Republic, with surgical treatment having been offered for more than 50 years. Patients may be on active TB treatment or may have completed TB treatment and have conditions such as post-TB aspergilloma, causing massive haemoptysis. They are usually referred from within the National TB Programme or from general practitioners outside the programme, or they may come self-referred.

There are five thoracic surgeons in Bishkek and three in Osh offering surgical treatment five days a week. Training to be a thoracic surgeon takes two to three years, which may include intensive short courses overseas in China. There is a special theatre in each hospital that is dedicated for thoracic surgery, with a team of 5–6 people (doctors and nurses) who are specialised in this type of surgery. The indications for surgery are clearly written in the national guidelines for MDR-TB treatment (Ministry of Health Kyrgyzstan. Clinical guidance on management of drug resistant tuberculosis. 2020). These are categorised as follows: emergency surgical treatment (haemoptysis and tension pneumothorax); urgent surgical treatment (steady progression of TB despite chemotherapy); planned surgical treatment (localised forms of destructive TB, tuberculoma); and surgical treatment as a result of complications and consequences of the TB process. Following surgery, patients are admitted to a post-thoracic surgical high-dependency unit for one day, followed by transfer to the general ward if the patients’ condition is stable.

The standard patient pathway is as follows. Patients are referred by their family doctors to a pulmonologist, and from there to a TB centre for diagnosis and treatment of TB. Clinicians at the TB centres refer patients to the thoracic surgical departments in Bishkek or Osh based on various criteria, which include prolonged and severe haemoptysis (defined as coughing more than 50–100 mL blood per day); round tumours on chest radiography; and non-response to medical treatment, combined with extensive cavitary disease.

A multidisciplinary approach is advocated, with anti-TB chemotherapy given before surgery, using at least four drugs for 6 months for DS TB (isoniazid, rifampicin, pyrazinamide and ethambutol) or second-line drugs for varying lengths of time for DR TB. Before surgery, patients undergo a full blood count and biochemistry analysis, screening for comorbidities, repeat mycobacterial microbiology if deemed appropriate, chest radiography and echocardiography. Depending on the circumstances, CT scans and bronchoscopy may also be carried out. Patients with confirmed TB after surgery are referred to the TB regional centres to continue TB treatment. Those with non-TB diagnoses after surgery have their TB treatment stopped and they are referred for appropriate medical management. Data on indications for surgery, surgical procedures and surgical outcomes are collected from hospital paper-based case records.

### 2.3. Study Population

The study population included, for Objective 1, all TB patients and all those who received thoracic surgery in the country between 2017 and 2021; and for Objective 2, TB patients who received thoracic surgery at Bishkek and Osh clinics in 2021.

### 2.4. Data Variables, Sources of Data and Data Collection

Data variables are shown in relation to the study objectives.

#### 2.4.1. For Objective 1 (Trend in TB Cases and TB Surgeries between 2017 and 2021)

Data variables included year, aggregate number of TB patients registered in the Kyrgyz Republic and aggregate number of TB patients receiving surgery in the two clinics (Bishkek and Osh). The sources of the data were WHO Global TB Reports, National TB Reports and Annual Reports on TB surgery from Bishkek and Osh clinics.

#### 2.4.2. For Objective 2 (TB Patients Who Received Surgical Treatment in 2021)

Data variables included sociodemographic and clinical characteristics, such as age, in years; gender; alcohol use; smoking status; migration status; HIV status; hepatitis status; body mass index (BMI); category of TB—new or previously treated; bacteriological status—bacteriologically confirmed or clinically diagnosed; drug susceptibility status—susceptible, resistant or not known; type of drug resistance—isoniazid mono-resistance, poly-resistance, RR/MDR or pre-XDR/XDR; disease location; date of starting anti-TB treatment; surgical parameters, such as date of surgical operation; indications for surgery; type of surgical procedure; diagnosis after surgery (TB or not TB based on medical report and/or ICCD coding); number of surgical operations; post-operative complications; surgical outcome (discharge from clinic, died, or absconded); and hospital discharge status; and TB treatment outcomes of those with confirmed TB after surgery, such as date of completing anti-TB treatment and treatment outcome—treatment success, died, lost to follow-up, failed or not evaluated. The sources of data for Objective 2 were individual paper-based patient records, the TB reports in both hospitals and the patient electronic database.

These data were extracted between January and April 2023.

### 2.5. Analysis and Statistics

Data were extracted to an EXCEL file and subsequently exported to EpiData Analysis version 2.2.2.186 (EpiData Association, Odense, Denmark) for further cleaning and analysis. Frequencies and proportions were used to describe characteristics and treatment outcomes. The chi-square test (with Fisher Exact test for small numbers < 5) was used to compare characteristics with TB treatment outcomes. The results were presented as unadjusted relative risks (RRs) and 95% confidence intervals (CIs). Levels of significance were set at 5% (*p* < 0.05)

## 3. Results

### 3.1. The Trend in TB Cases and TB Surgery in the Kyrgyz Republic, 2017–2021

**Figure 1:**
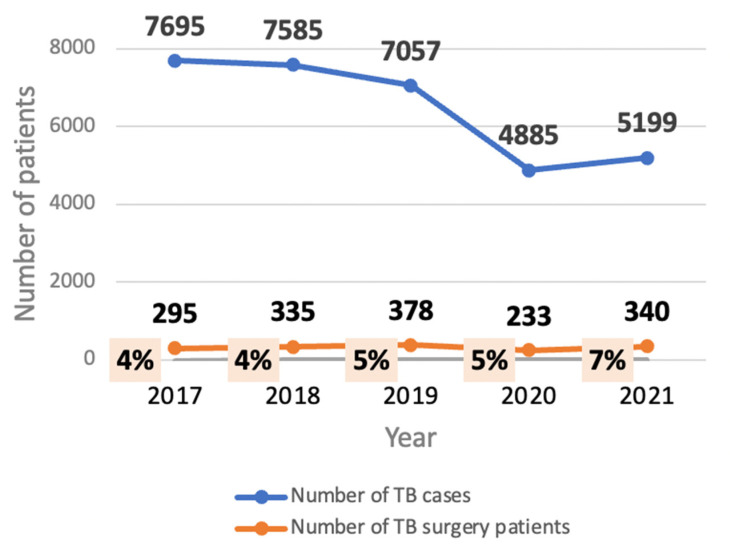
Trend in notified TB cases and TB patients undergoing thoracic surgery in the Kyrgyz Republic.

The trend in the number of registered TB patients in the Kyrgyz Republic and the number undergoing thoracic surgical treatment in Bishkek and Osh clinics between 2017 and 2021 is shown in Figure 1. There was a decline in the number of TB cases in the first four years and especially in 2020, at the time of the COVID-19 pandemic. Numbers increased slightly in 2021. The proportion of cases undergoing thoracic surgery remained fairly constant during this time, with a slight increase in 2021, varying from 4% to 7%. Over the five years, the types and proportions of radical surgical operations performed were as follows: pneumonectomy (5–10%); lobectomy (10%); segmentectomy (60%); pleurectomy, with or without decortication (10–20%); and others, including thoracoplasty, making up the remainder.

### 3.2. Characteristics of TB Patients Undergoing Thoracic Surgery in 2021

Case records were retrieved in 264 (78%) of the 340 patients with TB who underwent thoracic surgery in Bishkek and Osh in 2021. Their sociodemographic and clinical characteristics are shown in Table 1.

Nearly two-thirds of the patients were male. Less than 10% self-reported alcohol use or smoking. Just over half had normal nutrition based on their body mass index. In terms of comorbidity, no patient had HIV infection, 1% had serological evidence of Hepatitis B and 5% had serological evidence of Hepatitis C infection. Nearly two-thirds were new TB patients, and almost 80% were clinically diagnosed. Of 55 with bacteriological confirmation, 53% had drug-resistant disease, with RR/MDR being the predominant resistance pattern. Most of the patients had unilateral disease, with 10% having bilateral disease.

### 3.3. Timing of TB Treatment with Respect to Thoracic Surgery in TB Patients in 2021

**Figure 2:**
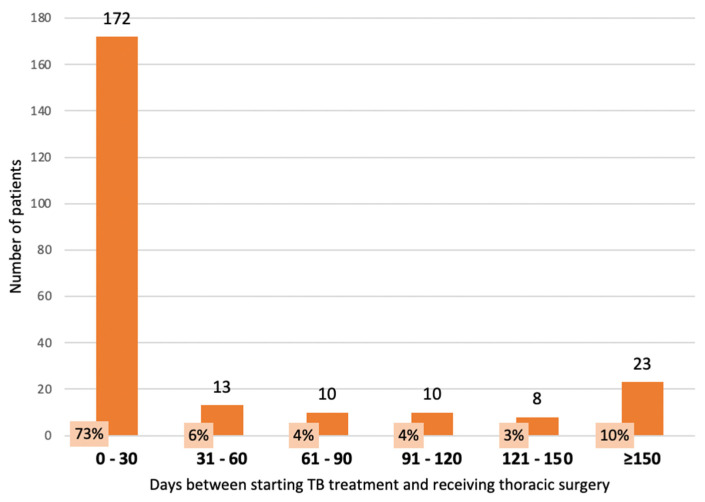
Timing of TB treatment before undergoing thoracic surgery in Bishkek and Osh, the Kyrgyz Republic, 2021.

Of the patients undergoing thoracic surgery, 236 (90%) patients started TB treatment before surgery; 22 (8%) had TB treatment after surgery; and for 6 (2%) patients, the dates were not recorded. The timing of TB treatment before surgery is shown in Figure 2. For those who had TB treatment before surgery, 95 (40%) had surgery within 0–6 days of starting TB treatment, 42 (18%) had surgery within 7–13 days and 35 (15%) had surgery within 14–30 days. For the 172 patients who had surgery within 30 days of TB treatment, 140 (81%) had clinically diagnosed TB. Of the 32 with bacteriologically confirmed TB, 16 (9.5%) had DS TB and 16 (9.5%) had DR TB. For those who started TB treatment after surgery, the median (interquartile range) number of days between the two events was 14 (5–91).

### 3.4. Details of Thoracic Surgery in TB Patients in 2021

The surgical details of TB patients undergoing surgery are shown in Table 2. Tuberculoma, pleural exudates and pleural empyema were the three most common indications for surgery, accounting for 76% of all cases.

Radical surgery was performed in 68% of patients, as follows. Those with tuberculoma mainly had a segmentectomy performed. Those with cirrhotic pulmonary/pleural disease or fibro-cavitary/cavernous disease mainly had a lobectomy performed, and sometimes pneumonectomy for severe disease. The type of surgery for those with haemoptysis, persistent bacterial expectoration (three patients with DR TB and two with DS TB) and caseous pneumonia depended on the underlying condition. Those with pleural empyema mainly had pleurectomy and repair of broncho-pleural fistula where indicated. There were 82 (31%) patients (those with pleural exudates) who had minimally invasive endoscopic surgery, with biopsy and surgical drainage to establish a diagnosis when this was in doubt and manage recurrent exudative effusions. Three patients underwent extra-pleural thoracoplasty.

Post-operative complications (recorded only for the first operation) were documented in 21 (8%) patients. The main complication was bronchopleural fistula, followed by post-operative pleural empyema and surgical skin infections. Twelve patients had post-operative complications following surgery for pleural empyema or pleural exudate, with nine patients having complications following surgery for tuberculoma and pulmonary disease. 

**Figure 3:**
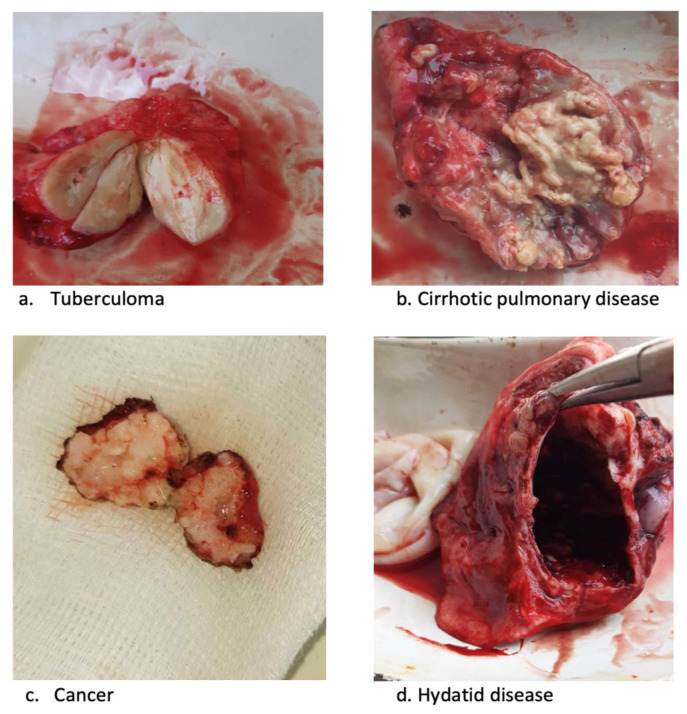
Resected specimens from thoracic surgery in Bishkek and Osh, the Kyrgyz Republic, 2021.

There were 201 (77%) patients who had TB confirmed at surgery, with 72% having just TB and 5% having both TB and non-TB disease at the same time (Table 2). There were 63 (23%) patients for whom TB was not confirmed at surgery. The most common specified histological non-TB conditions were cancer and hydatid disease, with miscellaneous conditions accounting for the remainder, as shown in the footnotes of Table 2. Altogether, 13/85 (15%) patients presenting clinically with tuberculomas were found not to have TB: the conditions were lung cancer in 4, hydatid cyst in 4 and another condition in 5 (specific condition not available in the case files). There were 39/82 (48%) patients with pleural exudates and 8/35 (23%) patients with pleural empyema who were found not to have TB on histology. In all patients in whom TB was not found after surgery, the TB treatment was stopped. Examples of some of the resected specimens are shown in Figure 3. The surgical outcomes were good. Overall, there was one death, and three patients reported no change in their condition upon discharge from the hospital. The one death occurred in a female patient with severe bilateral, fibro-cavitary, multidrug-resistant TB; her death occurred 4 months after surgery.

### 3.5. TB Treatment Outcomes and Factors Associated with Treatment Success

The TB treatment outcomes for the 201 patients with confirmed TB after surgery are shown in Table 3. Of these 201 patients, 163 (81%) of them were recorded as having treatment success (either cured or completed treatment). Six patients in total died or were lost to follow-up. The main unfavourable treatment outcome was “not evaluated” in 14% of the cohort.

The factors associated with TB treatment success are shown in Table 4. The only significant associations were a better treatment success in those undergoing surgery for tuberculoma (94%), for cirrhotic pulmonary/pleural disease (100%) and for haemoptysis (100%) compared with those undergoing surgery just for pleural exudate (56%). There were no significant differences with respect to other characteristics between those with clinically diagnosed TB and bacteriologically confirmed TB or between those with DS TB and DR TB.

## 4. Discussion

This is the first study in the Kyrgyz Republic that assessed the trend, characteristics and outcomes of TB patients undergoing thoracic surgery in the two principal thoracic surgical centres in Bishkek and Osh. There were five key findings that are of interest to the National TB Programme.

First, during the five-year period, 2017–2021, about 5% of registered TB patients in the country underwent thoracic surgery, with some minor variation from year to year. In 2020, with the onset of the COVID-19 pandemic, there was a sharp decline in the number of notified TB cases nationally. This was associated with a similar decline in the number of patients receiving surgery, although the proportion receiving surgery remained the same as in other years. The 2020 findings on case notifications are in line with those from elsewhere wherein presumptive and notified TB cases decreased drastically with the onset of COVID-19 [15,16,17]. There is limited information from other countries about what proportion of registered TB patients undergo thoracic surgery; however, our finding of 5% was less than the 10–12% found in neighbouring Uzbekistan [8].

Second, clinical advice [18,19] and national guidelines recommend starting TB treatment before surgery to reduce patient infectiousness and allow time for nutritional and immunological improvement to limit the risk of complications (Ministry of Health Kyrgyzstan. Clinical guidance on management of drug resistant tuberculosis. 2020). In our study, 90% of TB patients started TB treatment before surgery, with a substantial proportion undergoing surgery within the first month of starting TB treatment.

Did this pose an infectious risk for surgical staff? Conventional wisdom is that effective TB treatment results in a rapid decline in bacillary viability which quickly renders the patient less contagious to others: infectiousness decreases by over 90% by day 2 and by over 99% by day 14 to 21 [18]. Most of the early surgery performed in the first month was in patients with clinically diagnosed disease in whom infectiousness is less than in those with bacteriologically confirmed TB. Of the remainder who had bacteriologically confirmed TB, the numbers with DS TB and DR TB were approximately equal, and each of them was less than 10% of the total. We did not evaluate the risk to surgical and nursing staff, but it was unlikely to be that high provided that proper surgical procedures were followed, there was suitable face-masking of operating theatre staff and proper ventilation.

Did this early surgery pose a risk to patients? Nutritional status, as measured by body mass index, was satisfactory at the time of surgery, and there was little in the way of comorbidity or other risk determinants, such as smoking and alcohol abuse, that might have adversely affected outcomes. Furthermore, the surgical discharge outcomes were good, and there was only one death. Thus, overall, we believe that there was no significant risk to patients. We do not have information about what prompted early surgery, but the need to perform surgery as quickly as possible, if the patient has massive life-threatening haemoptysis, for example, will outweigh other considerations [20].

Third, nearly one-quarter of our patients had a diagnosis other than TB after surgery had been carried out, with cancer, hydatid disease and a range of other conditions masquerading clinically as TB. This finding is similar to the findings of other studies. For example, up to 15% of patients with clinically diagnosed pulmonary TB in Malawi were found to have another pathology [21]. In Uzbekistan, 20% of the TB patients undergoing surgery had a revised diagnosis, which was most commonly lung cancer, hydatid disease of the lung and post-TB fibrosis [8]. Establishing a histological diagnosis is an understated benefit of surgery. Exposing patients without confirmed TB to potentially toxic and expensive anti-TB drugs is harmful to the patient and wasteful of resources. Furthermore, in a region where hydatid disease of the lung appears to be common, individual patients diagnosed with this condition can be cured by surgery and anti-parasitic drugs.

Fourth, the surgical outcomes were excellent. Although we did not document the type of post-operative complications, these were recorded in less than 10% of patients, similar to what was found in Uzbekistan [8]. Most patients had an excellent response to surgery, with only one recorded death and only three patients experiencing no change in their condition at the time of discharge from the hospital.

Finally, TB treatment success in the cohort was satisfactory at just over 80%, with one death and small numbers being lost to follow-up or failing treatment. Previous studies amongst those undergoing radical surgery have reported that treatment success rates vary from 83 to 93%, with rates of 98% amongst those with DS TB [22,23,24,25]. The principal reason for an unfavourable TB treatment outcome in our study was the patient not being assigned a treatment outcome and therefore being recorded as not “evaluated”. In patients with confirmed TB, most baseline characteristics, determinants and comorbidities did not influence TB treatment success rates. However, having a tuberculoma, cirrhotic pulmonary/pleural disease or haemoptysis was significantly associated with better treatment success compared with having a pleural exudate. The likely explanation is that patients with a pleural exudate were not well followed up to the end of treatment and therefore not assigned a proper treatment outcome.

This study had several strengths. The study was endorsed by the National TB Programme in the Kyrgyz Republic as high priority, there was good reporting of aggregate trend data over five years, a large number of surgical patients were studied and the conduct and reporting of the study were line with the Strengthening the Reporting of Observational Studies in Epidemiology (STROBE) guidelines [26].

However, there were some limitations. First, in 2021, we could retrieve only 78% of the case records from the centres in Bishkek and Osh because many of these had been sent to a large case-record archive and could not be found. We, therefore, were unable to have a fully representative sample of thoracic surgical TB patients from the country for that year. Second, the definitions of some of our variables, such as self-reported smoking and alcohol use, were weak. Third, we had missing data for some variables. Fourth, we were unable to obtain precise numbers of different types of surgery, such pneumonectomy, lobectomy, segmentectomy and pleurectomy, or obtain precise information for non-TB diagnoses for each patient after surgery because the data had not been entered into the case records. Finally, we were not able to obtain country-wide data about why patients were selected for thoracic surgery, and we were not able to compare characteristics or outcomes of patients undergoing surgery with those not undergoing surgery. These were not objectives of our study and require a separate study to be undertaken, as the question is important. Although a previous systematic review and meta-analysis indicated that surgery as an adjunct to chemotherapy is associated with improved treatment outcomes in MDR-TB patients [4], these findings are not universal. A subsequent systematic review found no significant survival benefit of adjunctive surgery [24], and other studies found that different types of surgery (e.g., partial lung resection or full pneumonectomy) are important determinants of survival [27,28].

Despite these limitations, there are a few important implications and recommendations to be made. First, if the Kyrgyz Republic is interested in obtaining more specific data in the future on TB patients undergoing thoracic surgery, it is important to strengthen the monitoring database. The hospital case records should be restructured so that important information about the indications for surgery, different types of surgery, dates of starting surgery in relation to starting TB treatment and non-TB diagnoses is better recorded. For example, surgery for pleural exudates may be carried out for therapeutic and diagnostic purposes, and more precise recording details would allow for a better understanding of why the surgery is performed. Smoking and excess alcohol use are important determinants of TB treatment outcomes [29,30], so definitions need to be tighter and in line with international guidance.

Second, and as a follow-on from the first recommendation, the TB treatment outcome of “not evaluated” needs to be reduced to as close to zero as possible. “Not evaluated” often includes patients who are transferred out to another treatment unit and whose treatment outcome is not reported back to the initial registration unit [31]. The use of a mobile phone and other communication technologies can facilitate in reducing this correctable adverse outcome and improve treatment success rates.

Third, the good outcomes of TB patients undergoing thoracic surgery in the Kyrgyz Republic should lead to a reconsideration of the role of surgery in managing patients with this disease. Conventional wisdom is that patients with TB, especially those with bacteriologically confirmed disease, should only undergo thoracic surgery after several months of anti-TB treatment with chemotherapy to ensure that the patient is non-infectious and to allow nutritional and immunological improvement to occur. However, surgery may be needed earlier for complications, such as massive haemoptysis or failure to respond to anti-TB drugs due to extensive cavitation and destructive lung disease. This study shows that, despite early surgery, often within the first month of TB treatment, excellent surgical and TB treatment outcomes can be achieved. If surgery is performed early in the course of TB treatment, it will be important to ensure that front-line staff take appropriate infection and prevention precautions and that there is good ventilation in the operating theatres and the wards. Staff should consider wearing close-fitting N95 masks and using newer techniques, such as video-assisted thoracoscopic surgery, which results in shorter operation times and fewer post-operative complications compared with traditional thoracotomy [32].

## 5. Conclusions

In conclusion, this observational study in the Kyrgyz Republic showed that about 5% of TB patients in the country underwent thoracic surgery between 2017 and 2021. In 2021, most patients underwent thoracic surgery after being started on TB treatment, with a substantial proportion being operated on early within one month of TB treatment. Nearly one-quarter of patients did not have TB confirmed at surgery, with other common specified diagnoses being lung cancer and hydatid disease of the lung. The surgical outcomes were excellent. TB treatment success in those with confirmed TB was 80%, with the main adverse outcome being “not evaluated”. Moving forward, the Kyrgyz Republic needs to improve its monitoring and recording system to better capture variables and determinants that may affect surgical outcomes and to reduce the TB treatment outcome of “not evaluated”, which detracts from the overall TB treatment success. This study also shows that surgery was often performed early on in the course of treatment, in which case front-line surgical and nursing staff need to take appropriate infection prevention precautions to avoid being infected with *Mycobacterium tuberculosis* during the procedures.

## Figures and Tables

**Table 1:** Characteristics of TB patients in Bishkek and Osh, the Kyrgyz Republic, undergoing thoracic surgery in 2021.

Category	Variable	Number	(%)
Total	Total	264	
Gender	Male	161	(61)
	Female	103	(39)
Age in years	0–18	16	(6)
	19–39	163	(62)
	40–59	54	(20)
	≥60	21	(8)
	Not recorded	10	(4)
Alcohol use—self-reported	Yes	20	(8)
	No	227	(86)
	Not recorded	17	(6)
Smoking status—self-reported	Yes	20	(8)
	No	232	(88)
	Not recorded	12	(4)
Migration status	Internal/cross-border ^A^	14	(5)
	Not migrant	250	(95)
Body mass index (BMI)	Undernutrition (BMI < 18.5)	28	(11)
	Normal (BMI 18.5–24.9)	139	(53)
	Overweight (BMI 25.0–29.9)	36	(14)
	Obese (BMI ≥ 30.0)	10	(4)
	Not recorded	51	(18)
HIV status	Positive	0	-
	Negative	247	(94)
	Not recorded	17	(6)
Hepatitis B status	Positive serology	2	(1)
	Negative serology	238	(90)
	Not recorded	24	(9)
Hepatitis C status	Positive serology	13	(5)
	Negative serology	228	(86)
	Not recorded	23	(9)
Category of TB	New	161	(61)
	Previously treated	103	(39)
Bacteriological status	Bacteriologically confirmed	55	(21)
	Clinically diagnosed	209	(79)
Drug susceptible status ^A^	Susceptible	26	(47)
	Resistant	29	(53)
Type of drug resistance ^B^	Isoniazid monoresistance	4	(14)
	Polyresistance	2	(7)
	RR/MDR	20	(69)
	Pre-XDR/XDR	3	(10)
Disease location	Left unilateral	105	(40)
	Right unilateral	132	(50)
	Bilateral	27	(10)

Footnotes: migrant status—internal (13) and cross-border (1); ^A^ denominator = bacteriologically confirmed TB; ^B^ denominator = drug-resistant TB; RR = rifampicin-resistant; MDR = multidrug-resistant; XDR = extensively drug resistant TB.

**Table 2:** Surgical details of TB patients undergoing thoracic surgery in Bishkek and Osh, the Kyrgyz Republic, 2021.

Category	Variable	Number	(%)
Total	Total	264	
Presenting indications for surgery	Tuberculoma	85	(32)
	Pleural exudate	82	(31)
	Pleural empyema	35	(13)
	Cirrhotic pulmonary/pleural disease	22	(9)
	Fibro-cavitary/cavernous pulmonary	19	(7)
	Haemoptysis	14	(5)
	Persistent bacterial expectoration	5	(2)
	Caseous pneumonia	2	(1)
Type of surgical procedure ^A^	Radical	179	(68)
	Minimally invasive surgery	82	(31)
	Extra-pleural thoracoplasty	3	(1)
Number of surgical operations	1	252	(95)
	2	11	(4)
	3	1	(1)
Post-operative complications ^B^	Yes	21	(8)
	No	243	(92)
Diagnosis after surgery	TB only	189	(72)
	TB and non-TB together	12	(5)
	Non-TB only	63	(23)
Non-TB only ^C^	Lung cancer	15	(24)
	Hydatid disease of the lung	6	(10)
	Miscellaneous ^D^	42	(66)
Surgical outcome	Discharged	263	(99)
	Died	1	(1)
Discharge status	Good outcome after surgery	260	(99)
	No improvement after surgery	3	(1)

Footnotes: All variables are related to the first surgery; ^A^ radical procedures included pneumonectomy, lobectomy, segmentectomy and pleurectomy, while minimally invasive surgery included endoscopic biopsy of pleural tissue and removal of pleural fluid; ^B^ post-operative complications related to the first surgery that took place; ^C^ denominator is non-TB only; ^D^ miscellaneous = chronic respiratory disease with bullae, bronchiectasis, non-TB abscess, non-specific pulmonary/pleural inflammatory disease, non-TB empyema or pneumothorax, cystic lung disease and aspergilloma.

**Table 3:** TB treatment outcomes in patients in whom TB was confirmed as a result of thoracic surgery in Bishkek and Osh, the Kyrgyz Republic, 2021.

Category	Variable	Number	(%)
Confirmed TB		201	
Treatment outcomes	Treatment success	163	(81)
	Died	1	(<1)
	Lost to follow-up	5	(3)
	Failed treatment	4	(2)
	Not evaluated	28	(14)

Footnotes: The treatment outcome definitions are according to WHO guidelines [14]. Treatment success was defined as a TB patient being cured or completing a full course of TB treatment; lost to follow-up was defined as a TB patient who did not start treatment or whose treatment was interrupted for 2 consecutive months or more; failure was defined as a TB patient whose sputum smear or culture was positive at month 5 or later during treatment; not evaluated was defined as a TB patient for whom no treatment outcome was assigned.

**Table 4:** Factors associated with TB treatment success for patients in whom TB was confirmed as a result of thoracic surgery in Bishkek and Osh, the Kyrgyz Republic, 2021.

Category	Variable	Confirmed TB Patients	Treatment Success		RR	(95% CI)	*p*-Value
		n	N	(%)			
Total		201	163	(81)			
Gender	Male	130	107	(82)	1.04	(0.9–1.3)	0.55
	Female	71	56	(79)	ref		
Age group years ^A^	Age < 60	185	150	(81)	1.22	(0.8–1.9)	0.50
	Age ≥ 60	9	6	(67)	ref		
Alcohol use ^B^	Yes	15	13	(87)	1.08	(0.9–1.3)	0.85
	No	173	139	(80)	ref		
Smoking status ^C^	Yes	17	13	(76)	0.94	(0.7–1.2)	0.85
	No	175	142	(81)	ref		
Migrant status	Yes	9	7	(78)	0.96	(0.7–1.4)	0.99
	No	192	156	(81)	ref		
Body Mass Index (BMI) ^D^	Undernutrition (BMI < 18.5)	22	20	(91)	1.11	(0.9–1.3)	0.30
	Normal (BMI 18.5–24.9)	105	86	(82)	ref		
	Overweight (BMI 25.0–29.9)	26	19	(73)	0.89	(0.7–1.1)	0.31
	Obese (BMI ≥ 30.0)	6	5	(83)	1.02	(0.7–1.5)	0.99
Hepatitis C status ^E^	Hepatitis C-positive	12	9	(75)	0.92	(0.7–1.3)	0.79
	Hepatitis C-negative	175	143	(82)	ref		
Category of TB	New TB	104	83	(80)	0.97	(0.8–1.1)	0.63
	Previously treated TB	97	80	(82)	ref		
Bacteriological status	Bacteriological confirmed	51	39	(76)	ref		
	Clinically diagnosed	150	124	(83)	1.08	(0.9–1.3)	0.33
Drug susceptible status	Drug susceptible	24	17	(71)	ref		
	Drug resistant	27	22	(81)	1.15	(0.8–1.6)	0.57
Timing of anti-TB treatment	<2 months before surgery	132	102	(77)	ref		
	≥2 months before surgery	51	44	(86)	1.12	(0.9–1.3)	0.17
	Before surgery	183	146	(78)	ref		
	After surgery	18	17	(94)	1.18	(1.0–1.4)	0.22
Presenting surgical indications	Tuberculoma	72	68	(94)	**1.69**	**(1.3–2.2)**	**<0.001**
	Pleural exudate	43	24	(56)	ref		
	Pleural empyema	27	20	(74)	1.33	(0.9–1.9)	0.12
	Cirrhotic pulmonary/pleural	22	22	(100)	**1.75**	**(1.3–2.3)**	**<0.001**
	Fibro-cavitary pulmonary	18	14	(78)	1.39	(0.9–2.0)	0.18
	Haemoptysis	12	12	(100)	**1.72**	**(1.3–2.3)**	**0.01**
	Persistent expectoration	5	2	(40)	0.72	(0.2–2.2)	0.54
	Caseous pneumonia	2	1	(50)	0.90	(0.2–3.7)	0.99
Disease location	Unilateral	184	152	(83)	ref		
	Bilateral	17	11	(65)	0.78	(0.5–1.1)	0.07

Footnotes: data not recorded for confirmed TB patients: ^A^ = 7; ^B^ = 13; ^C^ = 9; ^D^ = 42; ^E^ = 15. RR = relative risk; CI = confidence interval. Bold indicates significant relative risk.

## Data Availability

Data in this study can be made available upon request by contacting the corresponding author at kuznetsova@aph.org.ua.

## Open-Access Statement and Disclaimer

In accordance with WHO’s open-access publication policy for all work funded by WHO or authored/co-authored by WHO staff members, WHO retains the copyright of this publication through a Creative Commons Attribution IGO license (http://creativecommons.org/licenses/by/3.0/igo/legalcode (accessed on 15 May 2023)), which permits unrestricted use, distribution and reproduction in any medium provided the original work is properly cited. There should be no suggestion that WHO endorses any specific organisation, products or services. The views expressed in this article are those of the authors and do not necessarily reflect those of their affiliated institutions. The use of the WHO logo is not permitted. This notice should be preserved along with the article’s original URL.
